# Diverse clinical manifestations and prognosis in a couple’s mercury poisoning caused by skin-lightening creams: two case reports and literature review

**DOI:** 10.3389/fmed.2024.1511493

**Published:** 2025-01-22

**Authors:** Huixia Ji, Ye Chen, Dandan Liu, Tongzhou Zhou, Yuhua Tang

**Affiliations:** ^1^Department of Occupational Disease, Nanjing Prevention and Treatment Center for Occupational Diseases, Nanjing, China; ^2^Department of Clinical Laboratory, Nanjing Prevention and Treatment Center for Occupational Diseases, Nanjing, China

**Keywords:** mercury poisoning, nephrotic syndrome, mercury removal therapy, prognosis, case report

## Abstract

Skin exposure to mercury-containing creams occurs most commonly in young and middle-aged women and, in a few cases, in men. This article presents the symptoms and prognosis of a couple who developed mercury poisoning after cosmetic use of similar duration and dosage. Case 1 is a 33-year-old man who developed nephrotic syndrome after using skin-lightening creams containing mercury over 9 months. Renal puncture pathology indicated membranous nephropathy. During the course of the illness, the patient intermittently took Chinese medicine. Approximately 4 months later, the patient developed pulmonary thrombosis and lower extremity venous thrombosis as a result of fatigue driving and had to undergo thrombolysis and filter implantation. A urine mercury level of 65.4 μg/g·creatinine was detected in the patient. The urine protein level remained positive 8 months after mercury removal. Case 2 is a 30-year-old woman, the wife of case 1, who used the same creams for 9 months with her husband and had a urine mercury level of 80 μg/g·creatinine. The patient experienced sleep disturbances, fatigue, and irritability. In Case 2, neurasthenia symptoms were relieved following mercury removal, and no other complications were observed. There have been very few reports regarding male patients developing nephrotic syndrome as a consequence of using cosmetics that contain mercury. However, clinicians should not neglect this cause when dealing with newly diagnosed male patients with nephrotic syndrome. The treatment and prognosis of male patients are less well established, and changes in their condition must be closely monitored.

## 1 Introduction

Mercury poisoning occurs as a result of exposure to mercury or its compounds through the respiratory tract, digestive system, or skin. The symptoms associated with mercury poisoning can range from mild allergic reactions to severe impairments of the nervous and renal systems (1). It has been reported that cosmetics account for approximately 70% of cases of mercury poisoning (2). Mercury (II) salts promote skin lightening by inhibiting tyrosinase, an enzyme crucial for melanogenesis, thereby reducing melanin synthesis. According to the World Health Organization, skin-whitening products should not contain more than one part per million (ppm) of mercury (3). However, skin-lightening cosmetics containing excessive levels of mercury remain widely available in numerous regions globally. Consequently, these products are a frequent cause of chronic mercury poisoning among women (4, 5).

It has been observed that the nervous system and kidneys are the organ systems most significantly impacted by chronic mercury poisoning. Chronic mercury poisoning exhibits diverse clinical symptoms, which are governed by the specific chemical forms, exposure doses, and individual susceptibility (6).

In recent years, the majority of studies have focused on the potential risks that mercury-containing cosmetics may pose to female patients. However, there is a significant lack of reports concerning mercury poisoning in males resulting from skin-lightening cosmetics. This article presents the clinical features of mercury poisoning observed in two young married couples who exhibited markedly different clinical symptoms, disease progression, and prognoses after using mercury-containing skin-lightening products at comparable doses and duration.

## 2 Case presentation

### 2.1 Case 1

A 33-year-old man weighing 90 kg, who had smoked 20 cigarettes a day for more than 10 years, had no family history of autoimmune disease and reported no proteinuria on previous medical examinations. Over the last 10 years, the patient has been employed as a hairdresser. Since January 2023, the patient has been using skin-lightening lotions and creams on alternate days for 9 months, which he bought from a beauty salon.

**Figure 1:**
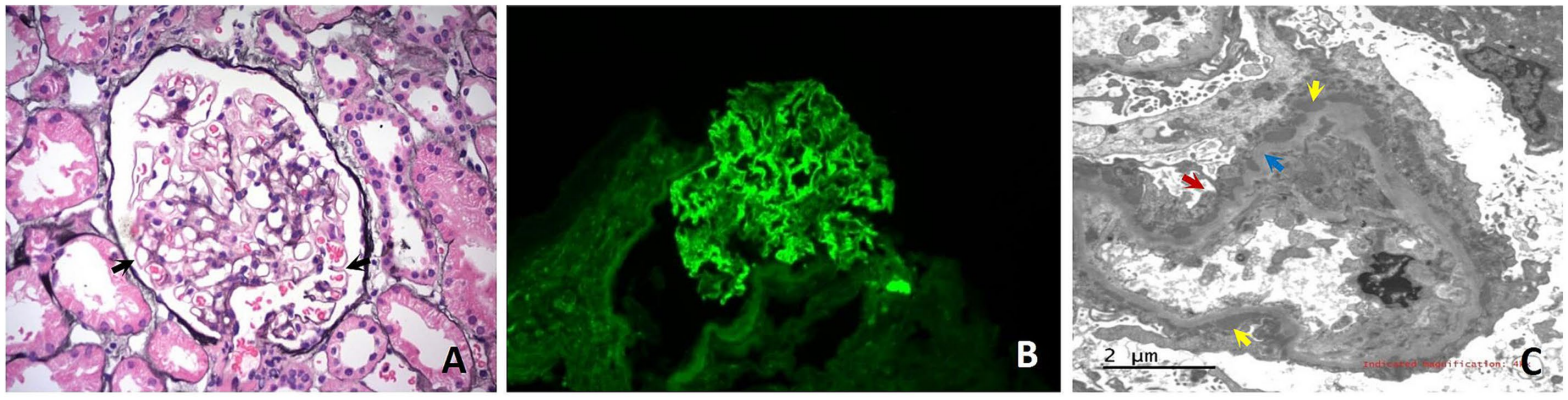
Histological images from the renal biopsy of Case 1. **(A)** Light microscopy shows glomerulus with the stiff capillary loops, thickening of basement membranes and formation of spikes (black arrows) (PASM × 400). **(B)** Immunofluorescence microscopy shows Immunoglobulin G deposits as fine granules along the capillary loops (×400); **(C)** Electron microscopy shows glomerular basement membrane irregularly thickened and diffuse fusion of podocyte foot processes (red arrows). Electron-dense deposits are noted beneath the epithelium (yellow arrows). Hyperplastic changes of the basement membrane are observed around some of the electron-dense deposits (blue arrows).

In September 2023, the patient developed edema of both lower limbs and foamy urine. Urinalysis revealed protein 2+, blood biochemical tests revealed albumin at 35 g/L, and creatinine levels were within normal limits in the initial hospital. After hospitalization, the patient underwent a renal puncture biopsy. The results showed that the patient had stage II membranous nephropathy, PLA2R and THSD7A were both negative (as shown in Figure 1).

He was then referred to the outpatient clinic of a teaching hospital in the provincial capital city, where he was prescribed valsartan and Chinese medicine without hormone therapy or immunosuppression. Initially, the patient took Chinese medicine intermittently but then stopped taking it.

The patient often worked overtime and stayed late because of his schedule. In January 2024 the patient developed chest tightness, cough, and hemoptysis after a 20-h long-distance drive. In February 2024 the patient was admitted to a local hospital for treatment. CTA of the pulmonary arteries showed multiple pulmonary embolisms in both the main trunk and branches of the bilateral pulmonary arteries. Pre-operative angiography revealed thrombosis-like manifestations in the right calf deep vein, popliteal vein, superficial femoral vein, common femoral vein, and the middle and distal segments of the right iliac vein. The deep vein of the left calf was not visualized, the left popliteal vein had a double-track sign, and the left superficial femoral vein had no middle or distal segments. Blood biochemistry analysis revealed an albumin level of 13 g/L. The patient underwent inferior vena cava filter placement, mechanical thrombectomy for the pulmonary artery thrombus, and contact thrombolysis with thrombolytic catheter placement. The patient underwent thrombolysis and anticoagulation therapy.

In March 2024 his condition was re-evaluated at a teaching hospital in the provincial capital. The serum albumin level was 28.8 g/L, the creatinine level was 83.6 μmol/L, and antiphospholipase A2 receptor antibodies (anti-PLA2R) were found to be negative. Additionally, the urine protein level was measured at 16.04 g/24 h. This patient’s treatment regimen includes calcitriol capsules 0.25 μg once daily, atorvastatin calcium 20 mg once daily, valsartan 80 mg once daily, rivaroxaban 20 mg once daily, tacrolimus 1 mg twice daily, and prednisolone acetate 10 mg twice daily. The patient was advised to undergo urinary mercury testing.

The patient underwent a urinary mercury test at our outpatient clinic on 8 March 2024, revealing a level of 65.4 μg/g·Cr (normal < 4 μg/g·Cr). In April 2024 on admission to our department, the patient still had foamy urine and edema of the right lower limb. The patient’s temperature was 36.7°C, his heart rate was 110 bmp (later reduced to 80 bmp), and his blood pressure was 124/73 mm Hg. Physical examination revealed no rash, white nail stripes (Mees’ lines), scaling of the hands or feet, or discoloration of the gums (mercury lines). Mild pitting edema of the right lower limb is observed. No abnormalities were observed upon neurological examination. Upon admission, routine blood tests revealed the following results: white blood cell count of 11.8 × 10^9^/L, neutrophil count of 67.8%, red blood cell count of 5.53 × 10^12^/L, hemoglobin level of 167/L, and platelet count of 275 × 10^9^/L. Urine analysis showed a protein level of 3+ and microalbumin levels ≥150 mg/L. The biochemical profile of the blood indicated a glutamyl transpeptidase level of 119 U/L, total protein concentration of 49.7 g/L, albumin concentration of 29.5 g/L, total cholesterol level at 9.16 mmol/L, triglyceride level at 1.72 mmol/L, HDL (high-density lipoprotein) at 4.22 mmol/L, LDL (low-density lipoprotein) at 5.58 mmol/L, glucose level at 5.22 mmol/L, urea concentration at 6.58 mmol/L, creatinine concentration at 80 μmol / L, and uric acid concentration at 445 μmol/L.

The patient was administered three courses of chelation therapy with 2,3-dimercaptopropane-1-sulphonate (DMPS) 0.25 g once daily for three consecutive days, followed by a 4-day interval. In the first course of treatment, the urinary mercury levels were 960 μg /24 h, 630 μg /24 h, and 319.8 μg /24 h, respectively. In the second course of treatment, the urinary mercury levels were 828 μg /24 h, 239.2 μg /24 h, 168 μg /24 h, respectively. In the third course of treatment, the urinary mercury levels were 171 μg /24 h, 184.5 μg /24 h, 84 μg /24 h, respectively. There were no adverse reactions during treatment.

Two weeks after the mercury removal, the patient’s right lower extremity was edema-free. Repeat the blood biochemistry test: glutamyl transpeptidase 95 U/L, total protein 39.8 g/L, albumin 23.6 g/L, total cholesterol 7.65 mmol/L, triglycerides 2.05 mmol/L, HDL 2.96 mmol/L, LDL 4.47 mmol/L, glucose 3.94 mmol/L, urea 5.71 mmol/L, creatinine 78 μmol/L, uric acid 421 μmol/L. Urine routine: protein 3+, microalbumin ≥150 mg/L. In May 2024 the removal of the lower extremity venous filter was conducted. In September 2024, the patient’s liver function was reexamined: glutamyl transpeptidase 35 U/L, total protein 64 g/L, and albumin 37 g/L. In December 9, 2024, the patient’s urine test indicated: protein 2+.

A sample of the patient’s cream was sent to a laboratory for testing, which showed mercury levels exceeding the upper limit of normal by 10,000 times.

### 2.2 Case 2

The wife of Case 1, a 30-year-old woman used the same skin-lightening cosmetics as in Case 1, with the same frequency and duration. Several months after using mercury-containing cosmetics, the patient complained of insomnia, fatigue, and irritability. Due to elevated urinary mercury levels in Case 1, Case 2 presented to our outpatient clinic in March 2024. She was advised to undergo hospitalization for mercury removal due to a urine mercury level of 80 μg/g·Cr. The patient underwent mercury removal at the general hospital where she worked. Urine protein levels were negative before treatment. Blood biochemistry revealed an albumin level of 46.3 g/L and a triglyceride level of 1.94 mmol/L. Detailed blood biochemical parameters are presented in Table 1. The treatment plan consisted of daily administration of DMPS 0.25 g plus 0.9% sodium chloride solution 500 mL intravenously for three consecutive days, followed by a four-day break, and a week later, for three courses. In the first course of treatment, the urinary mercury concentration levels were 141.7 μg/g·Cr, 88 μg/g·Cr, and 3352.3 μg/g·Cr, respectively. In the second course of treatment, the urinary mercury levels were 3312.3 μg/g·Cr, 2003.4 μg/g·Cr, 1198.6 μg/g·Cr, respectively. In the third course of treatment, the urinary mercury levels were 686.7 μg/g·Cr, 676.2 μg/g·Cr. There were no adverse reactions during treatment. After mercury removal treatment, her fatigue and insomnia improved.

**Table 1:** Symptoms, blood biochemical test results (before mercury removal treatment) and treatment of Case 1 and Case 2.

	Case 1	Case 2
Symptoms	Edema of lower limbs and foamy urine	Insomnia, fatigue, and irritability
Albumin, g/L	29.5 (40.0–55.0)	46.3 (40.0–55.0)
Globulin, g/L	20.2 (20.0–40.0)	29.2 (20.0–40.0)
Alanine aminotransferase, U/L	23 (9–15)	8 (7–40)
Aspartate aminotransferase, U/L	15 (15–40)	10 (13–35)
Total cholesterol, mmol/L	9.16 (<5.18)	4.47 (2.80–6.00)
Triglyceride, mmol/L	1.72 (<1.7)	1.94 (0.50–1.70)
Creatinine, umol/L	80 (57–97)	39.4 (41.0–73.0)
Urea, mmol/L	6.58 (2.8–7.2)	2.71 (2.60–7.50)
Uric acid, umol/L	445 (208–428)	278.6 (200.0–420.0)
Mercury removal treatment	IM, DMPS 0.25 g × 3 day, 3 weeks	IVP, DMPS 0.25 g × 3 day, 3 weeks

In both case 1 and case 2, the blood tests were conducted in different hospitals; therefore, the normal ranges for each indicator varied.

**Figure 2:**
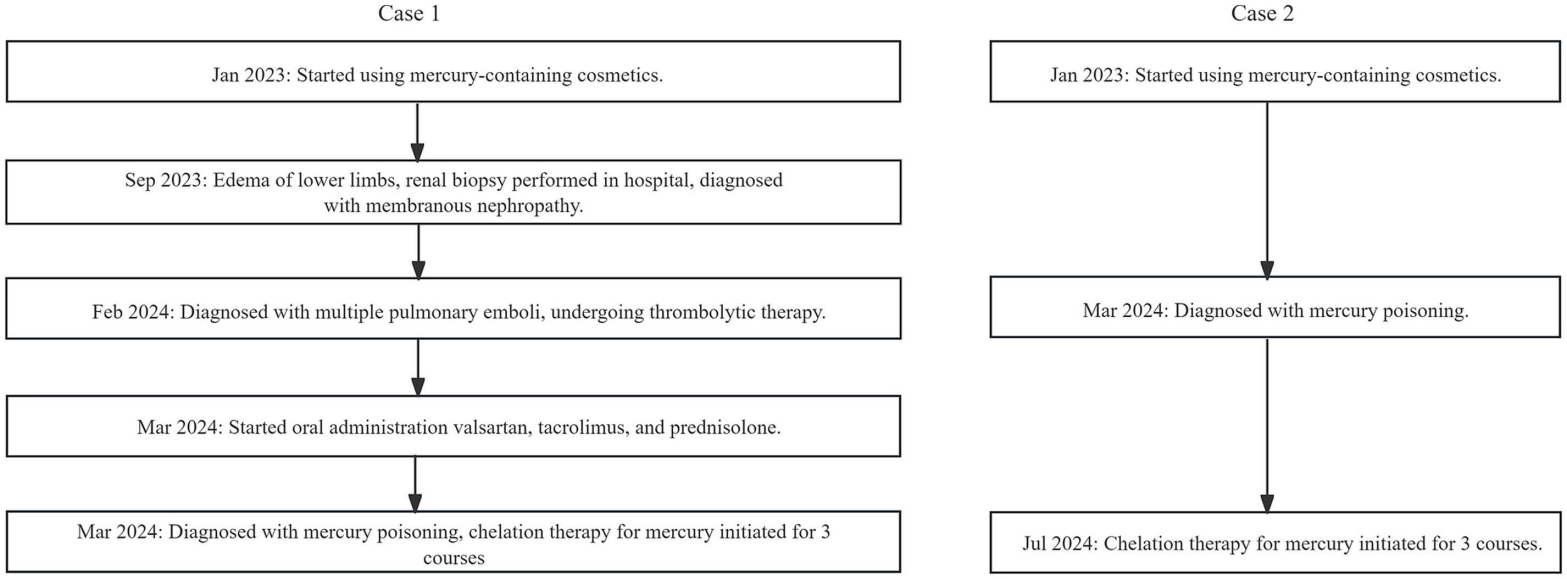
Flowchat of Case 1 and Case 2’s medical history.

**Figure 3:**
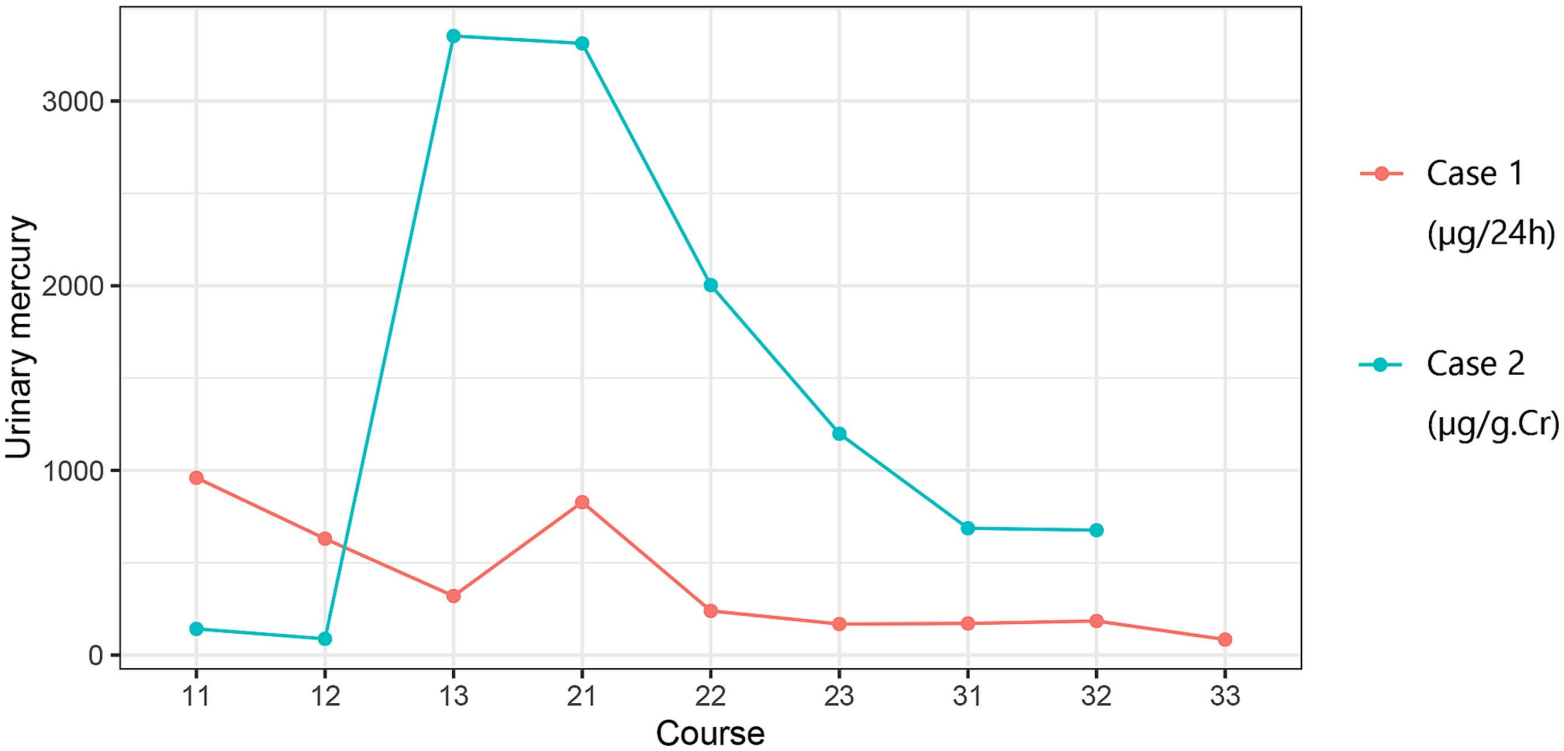
Results of mercury removal in Case 1 and Case 2. Course: 11: Week 1, Day 1; 12: Week 1, Day 2….

The medical history for case 1 and case 2 can be seen in Figure 2. Blood biochemical test results before mercury removal treatment of Case 1 and Case 2 were shown in Table 1. Figure 3 presents the total urinary mercury levels and concentrations collected daily for Case 1 and Case 2 throughout the mercury removal period. During the treatment of Case 1, the 24-h urinary mercury excretion was assessed after each mercury removal procedure performed in our hospital. During the treatment course of Case 2, mercury was removed in another hospital, and the concentration of urinary mercury was assessed following each procedure. During the third treatment course, Case 2 failed to provide a urine specimen the third intravenous infusion of DMPS. Upon a one-month follow-up of Case 2, the patient reported complete recovery of her health.

## 3 Discussion

Recently, cosmetic products containing excessive mercury have led to increased organ damage, particularly in the kidneys and nervous system (5, 7), owing to the desire for whitening. However, it is not certain whether male patients will have the same clinical characteristics and prognosis as female patients, as most patients in the current literature who have suffered mercury poisoning after using skin-ligntening cosmetics are female (4). In this study, the symptoms of individuals exposed to similar levels of mercury varied considerably. The male patient suffered nephrotic syndrome, pulmonary embolism, and lower limb thrombosis, whereas the female patient experienced only mild symptoms of neurasthenia.

The skin absorption rate is related to the mercury concentration and skin hydration status. Skin absorption is influenced by the skin’s integrity and the solubility of cosmetic carriers in lipids. Following absorption, inorganic mercury was distributed throughout all tissues. Due to the lipid solubility of mercury, it is readily permeable through the membrane and barrier of alveolar cells. Furthermore, it can permeate the blood–brain barrier and traverses the placenta by means of diffusion. A high concentration of mercury is present in the brain and kidneys. Within the kidneys, the concentration of mercury attains the highest level.

An expanding body of research has demonstrated that mercury, particularly in its inorganic divalent form, is linked to the development of autoimmune diseases (8). Activated T cells lead to the polyclonal activation of B cells, resulting in immune complex nephritis, such as membranous nephropathy, minimal change disease, or focal segmental glomerulosclerosis (9, 10). In Case 1, the mercury-related membranous nephropathy was caused by the use of skin-lightening cosmetics. Mercury-induced nephropathy remains inadequately understood. Upon the combination of mercury with proteins, haptens are generated, leading to the immune system’s production of antigen–antibody complexes. After infiltrating the glomerular basement membrane, these complexes induce glomerular lesions. In addition to exerting detrimental effects on the immune system, mercury can enhance the production of autoantibodies, inhibit T lymphocyte function, and trigger autoimmune disorders.

When a patient is diagnosed with mercury-related nephropathy, it is imperative that, in addition to mercury removal, comprehensive tests are performed, including renal puncture pathology, blood cholesterol, anti-M-type phospholipase A2 receptor (PLA2R), anti-thrombospondin type 1 domain 7 A receptor (THSD7A), and renal tubular function analysis. High levels of PLA2R and THSD7A expression have been observed in the kidneys of patients with idiopathic membranous nephropathy (IMN) and are associated with a poor prognosis (11, 12). THSD7A and PLA2R were not detected in the tissues of Case 1, suggesting a distinct immunological difference between mercury poisoning nephropathy and IMN. However, the patient failed to adhere to the recommended treatment, stopped taking the medication on his own, stayed up late, and drove fatigue for long periods, resulting in pulmonary embolism and lower-limb thrombosis. Therefore, patients with this type of nephrotic syndrome who are at risk of hypercoagulation should be closely monitored, and appropriate measures should be taken to prevent hypercoagulation.

Despite the poor permeability of the blood–brain barrier to mercury, prolonged exposure and slow elimination can lead to the accumulation of mercury ions in the central nervous system and cause neurotoxicity (8). When mercury ions interact with thiol groups, they lead to the formation of thiols. The thiol groups present in the brain play a crucial role in maintaining redox balance. Consequently, this reaction may disrupt cellular metabolism by inactivating thiolases. Furthermore, both *in vitro* and *in vivo* studies have demonstrated that exposure to mercury can induce oxidative stress (OS) (13), promote the generation of reactive oxygen species (ROS), and the depletion of glutathione (GSH) (14). As in Case 2, mercury damage to the central nervous system can manifest as insomnia, fatigue, weakness, mood swings, and other non-specific symptoms. The symptoms in Case 2 were significantly alleviated following mercury removal treatment.

Mercury poisoning should be treated with thiol-containing chelating agents. DMPS injections are the most commonly prescribed treatment. Both Cases 1 and 2 were treated with DMPS for mercury removal. The urinary mercury levels of both the husband and wife decreased after three courses of treatment.

The conditions and prognoses of patients with mercury-related nephropathy have also been studied. Mercury poisoning causes nephrotic syndrome, which is characterized by proteinuria, hypoproteinaemia, and hyperlipidemia but is less commonly caused by reduced renal function. Most mercury-related nephropathy cases have a good prognosis, with remission times ranging from 1 to 48 months and a median remission time of approximately 3 months. There was almost no recurrence after the mercury removal (6, 15). However, it is essential to note that most of these reports relate to young and middle-aged female patients, and there are relatively few reports on nephropathy in men who have used cosmetics containing mercury. After 8 months of follow-up, the urine protein in Case 1 remained positive for mercury-related nephropathy.

Men exhibit a higher susceptibility to occupational mercury poisoning, primarily due to their exposure to mercury vapor via the respiratory system in workplace environments (16, 17). Women are more likely to suffer from mercury poisoning due to the use of mercury-containing cosmetics. However, it remains unclear whether men are also at risk for mercury poisoning or related kidney disease from these products (18). Due to an irregular lifestyle and prolonged periods of staying up late, Case 1 discussed in this article may have a higher likelihood of developing kidney disease compared to mercury-poisoned individuals who maintain healthy working and resting habits. The rapid elevation in Ca2+ levels induced by testosterone induces calcium overload in cells, which contributes to mercury poisoning-induced kidney damage (19). Case 1 is a man who smoked for 10 years. Smoking also increases the risk of proteinuria (20). Smoking reduces nitric oxide (NO), which attenuates endothelial cell-dependent vasodilation and promotes intimal cell hyperplasia, leading to endothelial dysfunction and chronic kidney disease (21).

Additionally, high cholesterol levels contribute to poor prognosis in patients with membranous nephropathy. As plasma cholesterol concentrations increase, the cholesterol content in red blood cell membranes also increases, resulting in a decrease in fluidity, hindering oxygen diffusion, reducing the ability of red blood cells to load and release oxygen, and causing renal hypoxia. Another consequence of hypercholesterolemia is lipid deposition in the renal arteries, which can lead to a reduction in renal artery capacity and increased renal stress (22). At the time of hospital admission, Case 1’s plasma total cholesterol level was 9.16 mmol/L. Two weeks after receiving the mercury removal therapy, this level dropped to 7.65 mmol/L, indicating his health had improved.

Mercury-containing cosmetics were manufactured and used in Europe, Southeast Asia, Africa, the Mediterranean, and other regions from 2000 to 2022, whether purchased online or offline and recommended by friends and relatives, promoting the widespread use of whitening products and leading to mercury poisoning (23). Michael et al. reported that a 17-month-old female toddler developed hypertension, irritability, constipation, appetite loss, and leg pain after mercury exposure. The urinary mercury level was 243 mcg/g creatinine. The urinary mercury levels of her mother and grandmother after mercury exposure were 197 mcg/g creatinine and 222 mcg/g creatinine, respectively (normal 35 mcg/g creatinine). Still, they did not show any clinical symptoms. This suggests that individuals respond differently to exposure (24).

This study had some limitations. Case 2 was unable to undergo mercury removal treatment at our hospital due to work-related reasons, which led to a variation in the normal range of biochemical test results between Case 1 and Case 2. DMPS was injected intramuscularly in Case 1 and intravenously in Case 2. Although the treatment doses were the same, the different administration methods may have resulted in different mercury removal outcomes. As Case 2 only provided the urine concentration following mercury removal treatment, without providing the corresponding urine volume, it is not feasible to determine the amount of mercury excreted. Furthermore, patient 2 failed to provide a urine sample after the third injection of mercury removal in the third course of treatment.

Patients suspected of having mercury poisoning often require a multidisciplinary team that involves toxicologists and public health officials. To facilitate early recovery for patients affected by mercury poisoning, clinicians should strive to gain a comprehensive understanding of the clinical manifestations associated with this condition. Early identification and diagnosis are essential, as well as the removal of exposure sources and the implementation of chelation therapy. Furthermore, providing timely health education to patients is vital in managing their recovery effectively.

## 4 Conclusion

Generally, Nephrotic Syndrome caused by mercury-containing cosmetics have a better prognosis. Most of the research subjects reported in the literature on mercury poisoning caused by the use of skin-lightening creams are female patients. Whether male and female patients share similar clinical characteristics remains unknown. Some patients have a prolonged course of illness due to a lack of knowledge regarding mercury poisoning and heavy metal toxicity testing. Male patients may seek medical advice, but clinicians may overlook the possibility that mercury-containing cosmetics contribute to a range of complications. To reduce the number of misdiagnoses, it is imperative that the public is educated about mercury poisoning, including cosmetic use and occupational exposure. The significance of public health education in promoting the early detection of mercury poisoning cannot be overstated. Enhanced monitoring of mercury levels in cosmetics is crucial for mitigating mercury-related health risks and improving cosmetic safety.

## Data Availability

The datasets featured in this article are not readily accessible due to personal privacy. For inquiries regarding access to these datasets, please contact jihuixia1990@163.com.

## References

[1] Rodríguez-Viso P Domene A Sánchez A Vélez D Monedero V Devesa V. Challenges and strategies for preventing intestinal damage associated to mercury dietary exposure. Toxicology. (2023) 494:153580. doi: 10.1016/j.tox.2023.153580, PMID: 37328091

[2] Rojas-Franco P Franco-Colín M Torres-Manzo AP Blas-Valdivia V Thompson-Bonilla MDR Kandir S. Endoplasmic reticulum stress participates in the pathophysiology of mercury-caused acute kidney injury. Ren Fail. (2019) 41:1001–10. doi: 10.1080/0886022X.2019.1686019, PMID: 31736398 PMC6882499

[3] World Health Organization. Mercury in skin lightening products [Internet]. (2019). Available at: https://www.who.int/publications-detail-redirect/WHO-CED-PHE-EPE-19.13 (Accessed April 30, 2024).

[4] Chan TYK. Inorganic mercury poisoning associated with skin-lightening cosmetic products. Clin Toxicol (Phila). (2011) 49:886–91. doi: 10.3109/15563650.2011.626425, PMID: 22070559

[5] Ho YB Abdullah NH Hamsan H Tan ESS. Mercury contamination in facial skin lightening creams and its health risks to user. Regul Toxicol Pharmacol. (2017) 88:72–6. doi: 10.1016/j.yrtph.2017.05.018, PMID: 28554823

[6] Chan TYK Chan APL Tang HL. Nephrotic syndrome caused by exposures to skin-lightening cosmetic products containing inorganic mercury. Clin Toxicol (Phila). (2020) 58:9–15. doi: 10.1080/15563650.2019.1639724, PMID: 31314603

[7] Bastiansz A Ewald J Rodríguez Saldaña V Santa-Rios A Basu N. A systematic review of mercury exposures from skin-lightening products. Environ Health Perspect. (2022) 130:116002. doi: 10.1289/EHP10808, PMID: 36367779 PMC9651181

[8] Clarkson TW Magos L. The toxicology of mercury and its chemical compounds. Crit Rev Toxicol. (2006) 36:609–62. doi: 10.1080/10408440600845619, PMID: 16973445

[9] Becker CG Becker EL Maher JF Schreiner GE. Nephrotic syndrome after contact with mercury. A report of five cases, three after the use of ammoniated mercury ointment. Arch Intern Med. (1962) 110:178–86. doi: 10.1001/archinte.1962.03620200038008, PMID: 13866316

[10] Kumar MN Priyamvada PS Chellappan A Sunooj KV Srinivas BH Nachiappa Ganesh R. Membranous nephropathy associated with indigenous Indian medications containing heavy metals. Kidney Int Rep. (2020) 5:1510–4. doi: 10.1016/j.ekir.2020.06.015, PMID: 32954075 PMC7486185

[11] Nieto-Gañán I Iturrieta-Zuazo I Rita C Carrasco-Sayalero Á. Revisiting immunological and clinical aspects of membranous nephropathy. Clin Immunol. (2022) 237:108976. doi: 10.1016/j.clim.2022.108976, PMID: 35276323

[12] Li SS Tang DE Dai Y. Advances in antigens associated with idiopathic membranous nephropathy. J Formos Med Assoc. (2021) 120:1941–8. doi: 10.1016/j.jfma.2021.06.014, PMID: 34244038

[13] Augusti PR Conterato GMM Somacal S Sobieski R Spohr PR Torres JV. Effect of astaxanthin on kidney function impairment and oxidative stress induced by mercuric chloride in rats. Food Chem Toxicol. (2008) 46:212–9. doi: 10.1016/j.fct.2007.08.001, PMID: 17881112

[14] Shenker BJ Pankoski L Zekavat A Shapiro IM. Mercury-induced apoptosis in human lymphocytes: caspase activation is linked to redox status. Antioxid Redox Signal. (2002) 4:379–89. doi: 10.1089/15230860260196182, PMID: 12215206

[15] Li SJ Zhang SH Chen HP Zeng CH Zheng CX Li LS. Mercury-induced membranous nephropathy: clinical and pathological features. Clin J Am Soc Nephrol. (2010) 5:439–44. doi: 10.2215/CJN.07571009, PMID: 20089494 PMC2827581

[16] Webster PC. Not all that glitters: mercury poisoning in Colombia. Lancet. (2012) 379:1379–80. doi: 10.1016/s0140-6736(12)60582-0 22509527

[17] Calabrese EJ Iavicoli I Calabrese V Cory-Slechta DA Giordano J. Elemental mercury neurotoxicity and clinical recovery of function: a review of findings, and implications for occupational health. Environ Res. (2018) 163:134–48. doi: 10.1016/j.envres.2018.01.021, PMID: 29438899

[18] Yawei S Jianhai L Junxiu Z Xiaobo P Zewu Q. Epidemiology, clinical presentation, treatment, and follow-up of chronic mercury poisoning in China: a retrospective analysis. BMC Pharmacol Toxicol. (2021) 22:25. doi: 10.1186/s40360-021-00493-y, PMID: 33941274 PMC8091676

[19] Benten WPM Becker A Schmitt-Wrede HP Wunderlich F. Developmental regulation of intracellular and surface androgen receptors in T cells. Steroids [Internet]. (2002) 67:925–31. doi: 10.1016/S0039-128X(02)00055-7, PMID: 12234628

[20] Yoon HJ Park M Yoon H Son KY Cho B Kim S. The differential effect of cigarette smoking on glomerular filtration rate and proteinuria in an apparently healthy population. Hypertens Res. (2009) 32:214–9. doi: 10.1038/hr.2008.37, PMID: 19262485

[21] Orth SR. Effects of smoking on systemic and intrarenal hemodynamics: influence on renal function. J Am Soc Nephrol. (2004) 15:S58–63. doi: 10.1097/01.ASN.0000093461.36097.D5, PMID: 14684675

[22] Sodhi CP Phadke SA Batlle D Sahai A. Hypoxia and high glucose cause exaggerated mesangial cell growth and collagen synthesis: role of osteopontin. Am J Physiol Renal Physiol. (2001) 280:F667–74. doi: 10.1152/ajprenal.2001.280.4.F667, PMID: 11249858

[23] Lewis J. Global beauty Hazard: assessing mercury in skin-lightening products. Environ Health Perspect. (2023) 131:14002. doi: 10.1289/EHP12495, PMID: 36705937 PMC9881649

[24] Ori MR Larsen JB Shirazi FM. Mercury poisoning in a toddler from home contamination due to skin-lightening cream. J Pediatr. (2018) 196:314–317.e1. doi: 10.1016/j.jpeds.2017.12.023, PMID: 29395180

